# The dominance of introspective measures and what this implies: The example of environmental attitude

**DOI:** 10.1371/journal.pone.0192907

**Published:** 2018-02-15

**Authors:** Siegmar Otto, Ulf Kröhne, David Richter

**Affiliations:** 1 Otto-von-Guericke University, Magdeburg, Germany; 2 University of Hohenheim, Stuttgart, Germany; 3 German Institute for International Educational Research (DIPF), Frankfurt/Main, Germany; 4 Socio-Economic Panel Study, DIW Berlin, Berlin, Germany; Aligarh Muslim University, INDIA

## Abstract

The behavioral sciences, including most of psychology, seek to explain and predict behavior with the help of theories and models that involve concepts (e.g., attitudes) that are subsequently translated into measures. Currently, some subdisciplines such as social psychology focus almost exclusively on measures that demand reflection or even introspection when administered to persons. We argue that such a focus hinders progress in explaining behavior. One major reason is that such an exclusive focus on reflections results in common method bias, which then produces spurious relations, or in other words, low discriminant validity. Without the valid measurement of theoretical concepts, theoretical assumptions cannot be tested, and hence, theory development will be hampered. We argue that the use of a greater variety of methods would reduce these problems and would in turn foster theory building. Using a representative sample of *N* = 472 participants (age: *M* = 51.0, *SD* = 17.7; 54% female), we compared the validity of a classical introspective attitude measure (i.e., the New Ecological Paradigm) with that of an alternative attitude measure (i.e., the General Ecological Behavior scale). The latter measure, which was based on self-reported behavior, showed substantially better validity that we argue could aid theory development.

## Introduction

The central aim of *behavioral sciences* such as social psychology is to explain and predict behavior [1]. Thus, behavioral sciences have to produce knowledge (i.e., develop and test theories and models) that can be applied to explain and predict behavior. The next step in this quest is to develop consistent and parsimonious theories with testable models that consist of measurable concepts (e.g., attitudes, intentions, control beliefs), and finally, to explain or predict behavior. In order to increase explanatory power, researchers develop theories with the help of additional constructs that explain how attitudes influence behavior (for a broader overview on attitude research see [2]). However, the increases in the amount of variance that can be explained with each additional concept are often modest, and theory building suffers from **high interdependencies between explanatory concepts**, or in other words, **low discriminant validity** (e.g., [3]). These interdependencies could, of course, result from true correlations between the concepts. But what if they are nothing more than methodological artefacts that obstruct a clear view of the true interdependencies of the underlying theoretical constructs?

We argue that the method bias that often results from behavioral science’s strong reliance on introspective measures is a common critical methodological issue that deserves attention. Many measures in social psychology are based on participants’ verbal statements that require at least some degree of introspection. For example, participants have to reflect on what social norms are, reflect on their own intentions, recall their own values, or evaluate objects verbally. In contrast to other rather direct measures (e.g., attainment tests, observations, and reports of past behavior), introspective measures are more indirect because they involve evaluative and cognitive processes that are open to many more individual influences (e.g., self-presentation) and, thus, are much more prone to influences that are unrelated to the measured construct. If these influences are similar across different measures due to the use of the same method of measurement (e.g., introspective measures), common method bias can occur. We can only speculate which of the potential causes of method bias listed by Podsakoff and MacKenzie [4] have the strongest influence and distort the answers of introspective measures. But most likely common rater effects that help to present a desirable and consistent self (e.g., the consistency motif, social desirability, and implicit theories) probably have a strong influence on introspective measures as they demand introspection by design. In this respect, the evaluation of an object as requested by common attitude measures is a prototypical case of such introspective measures.

Thus, and because attitudes play a central role in explaining behavior [5, 6], we use the **concept of attitude to exemplify common method bias**. Furthermore, as presented in textbooks (e.g., [7]), the attitude concept provides a perfect platform for validation studies because attitudes can be expressed in different ways: A person can articulate a verbal statement that demands reflection (e.g., “The natural environment is important to me!”), express a certain emotion (e.g., laugh or cry), or act in a particular way (e.g., donate money to an environmental organization). Operationalizing all three forms of attitude expressions and using them in a methodologically more balanced way are the prerequisites of a more desirable heterogeneous measurement approach (i.e., multitrait-multimethod matrix; [8]). However, attitude research relies almost exclusively on introspective, or more specifically, evaluative verbal statements.

More specifically, **our study is based on measures of environmental attitude** of which a considerable number of different measures exist (e.g., [9]). Most such introspective measures that are used to predict ecological behavior can be divided into either specific measures of attitudes toward such ecological behaviors or more general attitudes toward the environment. One introspective measure of a general attitude toward the environment, that is, the New Ecological Paradigm (NEP), has been used extensively for more than two decades (e.g., [10]).

We were able to find only one corroborated environmental attitude measure that was not based on introspective verbal statements, that is, the General Ecological Behavior scale (GEB; e.g., [11]). We introduce the GEB as an example of a methodologically distinct behavior-based attitude measurement approach that, like the NEP, was designed to measure environmental attitude. By administering both measures to a representative sample, we demonstrate the differences the GEB brings to a setting dominated by introspective attitude measures. However, by no means are we claiming that either of the two attitude measures is inferior to the other measure in general. It is merely the ubiquitous use of introspective measures in some subdisciplines of psychology that we claim is problematic. Hence, our goal is to provide a discussion of how a more diverse mixture of methods will provide an opportunity to foster theory development, especially in the behavioral sciences. We achieve this goal by showing—with the help of our example from environmental psychology—how method bias can be reduced and how validity can be increased.

Even though our study focuses on strong empirical interdependencies between theoretically distinct concepts (i.e., low discriminant validity), the validation of measures with meaningful related criteria (i.e., **convergent validation**) is even more important for any measure. Thus, and in order to draw a more complete picture, we also test the convergent validity of our example measures. With respect to behavioral science, the concepts and their measures have to converge with meaningful criteria, which is foremost overt behavior. Thus, convergent validity plays a crucial role on a methodological level and seems to be a recurring problem. Especially in attitude research, convergent validity (i.e., correlations between attitudes and corresponding behaviors) is disappointingly low or even nonexistent (e.g., [12, 13]), which also might be a driver for researchers to develop models and theories with more concepts. As most of the measures of these concepts are introspective measures, this strategy potentially also fosters the problems of increasing numbers of barely distinguishable concepts and measures with low discriminant validity.

Overall, this study makes the argument that many of the interrelations of several introspective measures but also the discordance between introspective and behavioral measures are due to introspection, which leads to common method bias. Please note that we begin each of the following parts of the manuscript by exploring convergent validity, which is not the main focus of our study but is the more common and more important part with respect to validation. After exploring convergent validity, we get to the main concern of this study, that is, discriminant validity. We decided to use this order because it actually reflects the common order of validation studies even though our focus is on the second part.

### The dominance of introspective measures and the implications of such a practice

For more than two decades now, introspective measures have dominated social psychology and related fields [1]. Major reasons for the dominance of introspective measures are most likely that they are easier and more economical to use than most other measures. Especially in comparison with behavioral observations, which had been in favor several decades ago, introspective measures are more convenient and use significantly fewer resources. Furthermore, behavioral observations run the risk of being unethical or simply impossible to implement in certain circumstances [1].

But what is the downside of this focus on introspective measures? First, their low convergent validity with behavioral criteria is a common and widely acknowledged problem of introspective measures, especially attitude measures [12, 14]. From the perspective of a practitioners this also means that attitudes only explain a marginal share of behavioral variance as compared to other variables such as income [15]. In attempts to overcome this low convergent validity and practically marginal relevance, additional concepts have been added to explanatory models but with limited success. For instance, in environmental psychology, Hines and Hungerford [6] developed a model of responsible ecological behavior that included concepts such as attitudes, locus of control, personal responsibility, and knowledge. A number of further models or approaches that have attempted to explain ecological behavior with additional constructs have been developed (e.g., [5, 14, 16]). Most of these studies suffer from the problem that the concepts they have employed are often and substantially interrelated. If these interrelations could be accounted for by substantive variance, they could be discussed on a theoretical level and would subsequently lead to theoretical adjustments. But if common method bias due to the focus on introspective measures is prevalent and leads to low discriminant validity, it will be impossible to identify true interrelations on a conceptual level and advance theory. Thus, a closer investigation of discriminant validity appears to be warranted.

We argue that low discriminant validity in fields that focus on introspective measures is, to a certain degree, the result of this particular focus itself because applying several introspective measures in a study increases the likelihood of finding spurious relations due to common method bias [4]. This problem seems to be especially prevalent when attitude measures are used, where common method variance tends to be high [17]. As one consequence of common method bias, even if the true correlation between two attitude measures is zero, the two measures tend to show an average correlation of *r* = .23 [4], which, in technical terms, points to unsatisfactory discriminant validity. With sample sizes beyond 200, the chances that such correlations will be significant are high. As a consequence, building consistent theories is hampered by spurious findings that are the result of low discriminant validity caused by the instrument.

Most psychological measures can be sorted into certain categories of different measurement dimension of which we want to mention and discuss the most relevant one with respect to our study. An important distinction is the differentiation into trait- and state-based measures. Whereas state-based measures are sensitive to situational influences, trait-based measures are not and are much more stable over time. Thus, comparing measures that are more state dependent with more trait-related measures with the same validation items would be problematic because the situation present at the time of measurement could influence the results. Therefore, we selected two trait-based measures.

In order to exemplify how much could be gained from reducing the focus on introspective measures and by including other, more distinct measures, we compared the validity of two different kinds of measures: The NEP as a classical introspective attitude measure and the GEB as a measure that is based on self-reported behavior. As this behavior can be transitively ordered by its difficulty, it can be represented by a Rasch model. Whereas the NEP does not resolve the attitude-behavior gap (e.g., [10]), the GEB measures attitudes on the basis of a class of overt behaviors—implying an axiomatic relation between attitude and behavior [18]. We used domain-specific validation criteria to compare the construct validity of the two measures, and thus, their usefulness. We focused in particular on the distinctiveness of these measures from other concepts, or in other words, on their discriminant validity, which, if satisfactory, could help to support theory development by providing less spurious findings.

## Materials and methods

Ethical permission is provided by the Scientific Advisory Board of DIW Berlin.

### Participants

The research infrastructure Socio-Economic Panel (SOEP) at DIW Berlin established a longitudinal Innovation Sample (SOEP-IS; *N* = 3695, *M* = 51.6 years, *SD* = 17.8 years; 52.4% female) in 2012 for particularly innovative research projects. The topics addressed by the SOEP-IS are determined through a competitive refereed application process in order to identify the “best” research questions and their operationalization. The SOEP-IS data are available as open access data to the entire scientific community (scientific open access). Details on the sampling strategies, response rates, attrition, and representativeness of the sample can be found in [19]. All data were collected by a professional high-quality fieldwork organization (Infratest Social Research, Munich). The NEP was administered to 1,128 respondents as part of a larger module on *Just Sustainable Development Based on the Capability Approach*. The adaptive version of the GEB was administered to a randomly selected subgroup from this module, resulting in 474 valid cases. For 2 of these 474 cases no NEP measure was available. Thus, in order to have comparable power for testing the correlations of both the NEP and GEB and the validation criteria, we restricted our sample to the 472 respondents (*age*: *M* = 51.0 years, *SD* = 17.7 years; 54% female) for whom both the NEP and GEB were available.

### Instruments

#### The NEP

The authors of the NEP argued that environmentalism (in the 1970s) challenges our fundamental views of the relationship between humans and nature and reflects shifts in society’s dominant social paradigm [10]. Thus, the conceptualization of the NEP “focused on beliefs about humanity’s ability to upset the balance of nature, the existence of limits to growth for human societies, and humanity’s right to rule over the rest of nature” (see p. 427 in [10]). Overall, the NEP was designed to express an ecological worldview that includes a wide range of facets and renders it a trait-based measure [10]. However, across an impressive number of studies, the NEP has been established as a measure of environmental attitude or concern [10, 20].

In line with our criticism that is related to the strong focus and reliance on introspective measures, the construct validity of the NEP has been established with similar introspective measures such as support for pro-environmental policies, the perceived seriousness of world ecological problems, and the perceived seriousness of state and community air and water pollution, resulting in correlations of *r* = .57, .61, and .45, respectively. These correlations are all within the range that could be expected for good construct validity. Furthermore, criterion validity, that is, the correlation between the NEP and ecological behavior, has also been shown to be positive (*r* = .31; [10]).

The NEP we administered in our study was a German translation of the original English version [10] and was administered in the form of a Computer-Assisted Personal Interview. The 15 NEP items were rated on a 5-point Likert scale (*strongly agree to strongly disagree*) and consisted of eight statements expressing a pro-environmental orientation and seven statements reflecting a negative orientation toward the environment. An example item is “Humans are severely abusing the environment.” After recoding the items, a high NEP sum or mean score expressed a pro-environmental orientation. Given the limitations of coefficient-alpha as a reliability measure, we additionally used a SEM-based procedure to calculate its 95% confidence interval [21]. The reliability (coefficient-alpha) of the NEP in the current study (α = .76, 95% CI [.72, 0.79]) was comparable to the reliability identified in previous studies [10].

#### The GEB

The GEB was developed as a behavior-based environmental attitude measure, thus providing an alternative to introspective attitude measures [22]. It is based on the alternative conception of attitudes developed in the 1960s by DeFleur and Westie [23] who equated attitudes “… with the probability of recurrence of behavior forms of a given type or direction” (p. 21). The type or direction of behavior on the GEB is ecological behavior. Each single behavior, when performed, involves costs such as time, money, or loss of comfort. For example, recycling dead batteries costs time, donating money to an environmental organization costs money, and not using a car in inclement weather represents a loss of comfort. According to Campbell [24], costs are the expression of a situational threshold, or in technical terms, the difficulty of the behavior. The costs of a behavior are determined by the circumstances under which the behavior takes place. Thus, under similar conditions (i.e., the regulatory conditions in one country such as Germany), behaviors can be transitively ranked according to their difficulty independent of persons [11]. On the other hand, the stronger a person’s attitude, the more costs he or she will endure to show the related behaviors. Why would someone donate money to an environmental organization or ride a bicycle through rain and snow if she or he was not motivated to help the environment? The more costs a person endures in a behavioral domain, the higher his or her attitude must be (for a comprehensive explanation of the Campbell paradigm see [11, 18, 22]).

Several studies have provided support for the GEB’s convergent validity. In addition to studies that have demonstrated the criterion validity of the GEB [25], a known-group validity test has been performed as well by separating people who just arrived at work by either bicycle, public transportation, or car. Thus, using knowledge about the environmental friendliness of their mode of transportation (i.e., cycling and public transportation are more environmentally friendly than going by car), we implemented a known-group approach (see Study 2 in [26]). As expected, when assessed with the GEB, the environmental attitude of those commuting by car was significantly lower than that of the other two groups. Furthermore, the convergent validity of the GEB was supported by a study that showed the expected pattern of differential item functioning when comparing two distinct populations [27].

The GEB was an adaptive version, or a Computerized Adaptive Test in technical terms, that was based on an item pool of 50 Rasch-model-scaled items that have frequently been administered in previous nonadaptive paper-based versions [11]. The 50 items of the GEB provide reports about (un-)ecological behavior such as “I bring empty bottles to a recycling bin” of which 32 were rated on a 5-point Likert scale ranging from 1 (*never*) to 5 (*always*), and 18 were rated on a nominal scale (0 = no; 1 = yes). Because 19 of the self-reported behaviors were unecological, they were reverse coded. Furthermore, the 32 five-point Likert items were converted to a dichotomous response format as suggested by Kaiser and Wilson [11].

During a Computer-Assisted Personal Interview, the SOEP-IS’s standard procedure for data collection, we administered the adaptive GEB scale on a computer by presenting one item per screen and assuming that the item parameters for the adaptive version were identical to previous paper-based versions. Based on item parameters (i.e., item difficulties) of the previously generated Rasch-model the adaptive item selection was implemented to maximize the Fisher Information criterion, and a maximum likelihood estimator was used to estimate the person parameters. Specifically, the first item was selected randomly out of the best five items for an initial person parameter of 0, and all of the following items were selected out of the best three items as exposure control (randomesque approach; [28]). Testing was terminated if either the estimated asymptotic standard error of the maximum likelihood estimate fell below 0.5 or the number of items answered exceeded 25. Respondents were allowed to skip items. The resulting test length was 19.2 items on average and was never shorter than 17 items. The average of the standard errors, which were derived from the adaptive GEB and based on the item parameters used for the Computerized Adaptive Test, was 0.53.

#### Validation items

The validation items consisted of three self-reported overt behaviors and two behavioral intentions that expressed an environmental attitude. These items were rated on a 5-point Likert scale except for the car ownership question, which was a dichotomous item (yes/no). Furthermore, the validation items consisted of 16 statements about the importance of life aspects that were developed in a project on sustainability [29] specifically for the SOEP-IS. In attitude research, such evaluative statements are commonly used as measures of values [30]. These statements were answered on an 11-point Likert scale ranging from 0 (*absolutely unimportant*) to 10 (*absolutely important*). Our categorization of the 16 aspects of life as convergent or discriminant items was based on the corroborated finding that environmental attitude is positively related to biospheric-altruistic value orientations, negatively related to egoistic values, and unrelated to other values [31]. Two of the life aspects expressed an environmental attitude, three expressed egoistic values (i.e., an orientation that conflicts with an environmental attitude), and 11 life aspects were expected to be unrelated to environmental attitude, thus forming the main goal of our study to test and compare discriminant validity. For instance, striving to obtain an income is a central egoistic value, whereas the need for economic stability does not fit the definitions of egoistic values or biospheric-altruistic values.

### Procedure

In order to compare the validity of the NEP and GEB in our study, we related each scale to the validation items. The validities of the two environmental attitude scales were assumed to increase as their correlations with the five ecological behaviors and intentions increased. Thus, for a high validity, we expected positive relations of the NEP and GEB with the pro-environmental life aspects and no significant correlations with the 11 unrelated life aspects. We calculated a simple validity score for each of the instruments by adding 1 point for all correlations that were in the expected direction (marked with a “+” in Table 1) and subtracting 1 point for all correlations that were not in the expected direction for the convergent items (marked with a “-” in Table 1). For the discriminant statements, we added 1 point for a nonsignificant correlation and subtracted 1 point for a significant correlation. The last column includes a comparison of correlations from dependent samples according to Eid and Gollwitzer [32].

**Table 1:** Correlations of the NEP and GEB with the validation items.

	*n*	GEB		NEP		*p*
**Overt behavior and behavioral intentions (convergent)**		r	score	r	score	(diff r)
Not owning a car	231	.056	0	-.236***	*-1*	**< .001**
Frequency of abstaining from using a car and using alternative modes (i.e., public transportation, cycling, or walking) instead	176	.270***	+1	.051	0	**.01**
Intention to abstain from car use in the future and intention to use alternative modes (i.e., public transportation, cycling, or walking)	176	.232**	+1	.084	0	.059
Frequency of buying organic products	289	.373***	+1	.217***	**+1**	**.013**
Intention to buy organic products	291	.365***	+1	.226***	**+1**	**.024**
**Cognitive statements expressing the importance of the respective domain**						
Convergent statements						
Protecting the environment	472	.165***	+1	.157***	**+1**	.445
Sustaining living conditions for future generations	469	.123**	+1	.124**	**+1**	.493
Negative convergent statements						
Income	471	-.147**	+1	-.094*	**+1**	.181
Career	447	-.169***	+1	-.117*	**+1**	.191
Fast individual travel	472	-.103*	+1	-.022	0	.083
Discriminant statements						
Family	472	**-.029**	+1	**.031**	**+1**	.154
Friends	470	**.002**	+1	**-.024**	**+1**	.329
Dwelling	471	**-.065**	+1	**-.042**	**+1**	.348
Health	471	**-.050**	+1	.134**	*-1*	**.001**
Safe neighborhood	472	**.009**	+1	**-.004**	**+1**	.412
Education and information	471	**.023**	+1	.095*	*-1*	.11
Economic stability	472	**-.035**	+1	.107*	*-1*	.008
Influence on political decisions	468	.107*	*-1*	**.020**	**+1**	.07
Religion	470	**.079**	+1	-.154**	*-1*	**< .001**
Social peace	470	**.041**	+1	**.075**	**+1**	.281
Freedom	470	**.030**	+1	.103*	*-1*	.107
Overall Score (maximum = 21)			**18**		**6**	

*Note*. Coefficients in bold indicate correlations that supported the validity of the instruments. Coefficients in italics indicate correlations that were contrary to our expectations. The substantially lower numbers of cases (n) in the first category of validation items (Overt behavior and behavioral intentions) results from an only partial overlap of the subsample to which the GEB was administered and the subsample that included these items.

*p < .05.

**p < .01.

***p < .001.

## Results

The mean for the NEP score was *M* = 3.81 (scale range 1–5; *SD* = 0.51), which replicated the common finding of a strong endorsement of the NEP [10]. The mean for the GEB was *M* = 0.07, *SD* = 0.80 (Logits), and the NEP was positively correlated with the GEB (*r* = 0.19, *p* < 0.001). The NEP was positively correlated with only two and even negatively correlated with one of the five ecological behaviors and intentions (see Table 1). The GEB was correlated with four of the five behaviors and intentions in the expected way. Four of the five convergent cognitive statements about life aspects were positively related to the NEP, whereas all five of them were positively related to the GEB. Please note that correlations below .3 are usually interpreted as small effects, whereas correlations of .3 or higher are considered good [33]. Table 1 shows that only two of the convergent validation items had correlations with the GEB above .3. However, the magnitudes of the correlations between the predictors of ecological behavior such as the NEP and GEB with actual behavior were usually in the lower ranges. One reason is the broad and heterogeneous class of behaviors that are considered ecological behaviors. Thus, within the domain of ecological behavior, the correlations of the GEB as a general measure of environmental attitude with the convergent validation behaviors were relatively substantial [13].

Furthermore, the NEP was correlated with five of the 11 discriminant cognitive statements about life aspects (e.g., the statements about the importance of health and religion), whereas the GEB was correlated with only one of the 11 discriminant cognitive statements (see Table 1). On the basis of our scoring system, the NEP scored six out of 21 points, whereas the GEB scored 18 out of 21. As an additional measure, we included a test to compare the correlations from dependent samples [32]. Using this test, we found six significantly different correlations and four more marginally significant different correlations that all indicated differences in favor of the GEB.

The GEB includes two items (i.e., car use and buying organic food) that are similar in content to the convergent validation items. In order to account for this fact and to show the robustness of our findings, we ran an additional Rasch analysis without these two items. As expected, the new person scores were almost perfectly correlated (r = .98) with the person scores from the original adaptive test. Also as expected, the correlations between the person scores that were based on the new 48-item version and the validation items showed exactly the same pattern as the original results in Table 1.

## Discussion

Our exemplary comparison between an introspective attitude measure (i.e., the NEP) and a behavioral attitude measure (i.e., the GEB) revealed two major weaknesses of the introspective attitude measure. First, our data showed low convergent validity for the NEP, thus replicating the commonly identified, often discussed, and never solved attitude-behavior gap that has been linked to classical introspective attitude measures. Second, but most important for this study, our results indicated low discriminant validity for the NEP, thus referring to a common method bias that has been shown in particular for introspective attitude measures [4]. Even though our competitive behavior-based attitude measure (i.e., the GEB) also showed some weaknesses because it did not show a perfect validation pattern, it nevertheless showed significantly better convergent and discriminant validity than the introspective attitude measure. By employing a widely used introspective attitude measure (i.e., the NEP), we were able to provide a solid replication of the problems (attitude-behavior gap and common method variance) involved in the use of introspective measures in general. Beyond the methodologically undesirable focus on just one measurement approach (e.g., [4, 8]), the corroborated gap between introspective attitude measures and behavior remains an unresolved problem for introspective attitude measures [34].

However, aspects of convergent validity—as the foremost aim of validation—have already been discussed widely elsewhere (e.g., [12, 14]). In this study, we focused more on **discriminant validity** as an important basis for distinctive measures that help to foster consistent and cumulative theoretical progress. Without discriminant validity, concepts are hard to distinguish. This can hinder the development of models with interrelated concepts because concepts become interchangeable, and model development that is based on these concepts becomes more arbitrary. By testing and comparing the discriminant validity of an exemplary introspective measure of environmental attitude with another environmental attitude measure that is not based on introspection, we found some evidence for common method bias as a cause for the low discriminant validity of widely used introspective measures. Even though we have no empirical evidence for which of the causes of the method bias were at work in our study, we argue that the most likely sources are common rater effects (e.g., the consistency motif, social desirability, and implicit theories) because they help to present a desirable and consistent self [4]. The reason for this might lie in the fact that introspective measures are much more prone to desirable and consistent self-presentation because they rely on evaluative and cognitive processes that easily allow for it. Thus, independent of the latent construct, the motive to consistently represent one self might lead to an equalization of answers on introspective measures, thus resulting in a measurement component that leads to common method bias and to low discriminant validity.

Low discriminant and convergent validities are especially problematic when introspective measures are used to predict overt behavior in applied research or when such measures are used in costly panel studies (e.g., the SOEP) that aim to provide meaningful information to a broad scientific community. Thus, even at this basic methodological level of measurement validity, it seems worthwhile to work on developing measures with significantly better discriminant validity and to consider more than just convergent validity. This is especially important in the field of environmental psychology because environmental attitude is frequently used to predict ecological behavior (for an overview, see [14]).

At least in the domain of environmental attitude, we were able to identify an alternative measure that was based on the Campbell paradigm, that is, the GEB. Beyond the promising results of our rather basic comparison study on construct validity, other studies using the GEB have already provided results on its good external validity with respect to actual behavior [22, 35, 36]. Even though the GEB is not based on independently observed behavioral responses and is instead based on self-reported behavior, its outcome corresponds well with actual behavior and even the ecological impact of the behavior [37]. More specifically, its criterion validity has been shown with an ecologically strong criterion on the individual level, that is, electricity consumption [25]. Furthermore, because the Campbell paradigm is based on the Rasch model, all the benefits that come with the Rasch model, such as adaptive testing, can be used and applied (e.g., [38]). For instance, the possibility to link two test of the same construct with only a few items that overlap, repeated measurement becomes much more convenient (e.g., [35]).

Using only single validation items that we correlated with our two measures is certainly a **limitation of our study**. It is not possible to calculate Cronbach’s alpha for single-item measures, and thus, we were not able to report the reliability of our single-item measures. Furthermore, it is common practice in psychometrics to assume that measures based on more items usually produce more reliable results than measures with fewer items (all else being equal). However, this view has been contested, and single-items measures are often quite acceptable measures as well [39]. Furthermore, other validation criteria such as observed behavior would strengthen our study further.

Finally, we would like to outline **a way to shift from introspective measures to more diverse measurement**. We showed that introspective and behavior-based items possess different response properties, which lead to different outcomes with respect to validation criteria. However, they are linked with respect to content when they refer to the same attitudinal object because each kind of item legitimately represents one aspect of the attitude concept, respectively (e.g., [7]). A person can articulate his or her attitude with a verbal statement (i.e., introspective item) or behave in a particular way and report this behavior (i.e., behavior-based item). Furthermore, according to the Campbell paradigm, articulating a verbal statement can also be interpreted as a performance on the same scale as the performance of self-reported ecological behaviors from the same attitudinal domain (e.g., ecological behavior). Thus, theoretically, it is possible to use both introspective and behavior-based items in the same scale. However, an empirical test needs to be applied to determine whether and how well this works for any set of items. Indeed, it has been shown in a methodological study that introspective and behavior-based items that address attitude toward the environment can be scaled together reliably [40].

The technical key to testing the fit of different items empirically—and thus to exchange introspective items for behavior-based ones—is the Rasch model. The ability of the Rasch model to utilize different sets of items to measure the same constructs (e.g., [35]) enables the researcher to adapt and optimize item sets without losing comparability across the different item sets. That is, a set with a focus on introspective items and a set that focuses on behavioral items that both refer to the same attitudinal object can be linked with only a few overlapping items. Technically, linking two such scales is a common and straightforward procedure that is described in textbooks and articles on Item-Response-Theory of which Rasch models are a part (e.g., [41, 42]). By exchanging introspective items for behavior-based items, reducing the common method bias is possible and enables a more diverse measurement on the item level.

Our exemplary comparison showed that the present focus on introspective measures might hinder the progress that is needed in theory development by producing spurious findings and that shifting toward more diverse measurement methods might ease this burden—at least in environmental psychology. However, environmental psychology is an applied subdiscipline of social psychology, and as such, it provides a typical example of a behavioral science that overuses introspective measures. Thus, the chances are good that our proposal to shift the focus from introspective measures to more diverse measurement methods will be useful in all behavioral sciences that are currently dominated by introspective measures.
